# How hindrance stress, proactive personality, and the employment relationship atmosphere affect employees’ innovative behavior

**DOI:** 10.3389/fpsyg.2022.969013

**Published:** 2022-10-11

**Authors:** Jianpeng Fan, Yukun Fan, Lingli Yu, Shuyu Man

**Affiliations:** ^1^School of Economics Management, Pingdingshan University, Pingdingshan, Henan Province, China; ^2^School of Economics and Trade, Henan University of Technology, Zhengzhou, Henan, China; ^3^School of Business Administration, Henan University of Animal Husbandry and Economy, Zhengzhou, Henan, China

**Keywords:** hindrance stress, proactive personality, employment relationship atmosphere, employees’ innovative behavior, hierarchical linear model

## Abstract

Hindrance stress is a stimulus factor in the workplace that has a certain impact on the innovative behavior of employees. Most existing studies focus on the analysis of individual-level factors, ignoring the important role of organizational-level factors. This study uses multiple linear models to empirically analyze the interaction mechanisms among hindrance stress, proactive personality, employment relationship atmosphere, and employee innovative behavior factors in the workplace. This study found the following: (1) Hindrance stress negatively affects employees’ innovative behavior. (2) A proactive personality positively affects employees’ innovative behavior. (3) A proactive personality plays a moderating role in the relationship between hindrance stress and employees’ innovative behavior. (4) The employment relationship atmosphere has a positive impact on employees’ innovative behavior. (5) The employment relationship atmosphere plays a moderating role in the relationship between hindrance stress and employees’ innovative behavior. This study enriches theoretical knowledge in the field of human resources and provides guidance for business managers on the effective encouragement of employees’ innovative behavior.

## Introduction

At present, with increasing complexity and uncertainty in the external environment, the innovative behavior of employees has become an important determinant of organizational performance improvement and longevity of organizations (Wang et al., 2019), which has prompted scholars to think about approaches for stimulating innovative behavior among employees (Inam et al., 2021). A review of the literature shows that exploration of the antecedents of employee innovation behaviors is mainly based on three types of behavior: (1) work characteristics, including work autonomy (Wu et al., 2014), work objectives, work requirements (Shin et al., 2017), and other factors; (2) individual factors, including personality traits (Madrid et al., 2014), goal orientation (Janssen and Yperen, 2004), self-concept (Grosser et al., 2017), motivation (Yuan and Woodman, 2010), and other factors; (3) the social situation, including leadership and supervisory behavior (Su et al., 2017), impartiality (Janssen, 2010), organizational climate (Yu et al., 2013), and other factors. Although a large number of studies provide a rich context for understanding the generation of employee innovation behavior, most studies examine the influencing factors of employee innovation behavior at an individual level, with a lack of in-depth research on situational factors and their influencing mechanisms at a team level (Zou et al., 2018). In addition, most existing studies focus on the analysis of single-level factors, while ignoring the important role of multi-level factors.

At present, most research on job characteristics focuses on the promotion effect of high-quality work resources and effective work environment on employees’ innovative behavior, but the prediction effect of workplace stressors on employees’ innovative behavior has not been paid enough attention (Anderson et al., 2014). Work stress is a stimulus factor in the workplace and is affected by elements such as work characteristics, organizational role, interpersonal relationships, and career development (Cavanaugh et al., 2000; Ren and Zhang, 2015). Stressors can translate into positive or negative outcomes based on the extent to which the stressors promote or hinder potential gains; these are known as challenge and hindrance stressors (Jiang et al., 2020). Among them, hindrance stress refers to work stress that limits employees’ abilities or impedes their work tasks (Zhao and Yang, 2020). Most scholars have discussed challenging stress and hindrance stress as a unified concept (Lu et al., 2011; Zhang and Guo, 2011). In recent years, although some scholars have subdivided pressure into challenge stress and hindrance stress (Zhang et al., 2016; Kronenwett and Rigotti, 2020; Inam et al., 2021; Yang and Li, 2021), relatively few studies have taken hindrance stress as antecedent variable alone, and the underlying mechanisms have not been explored in depth (Anderson et al., 2014). In view of this, this study attempts to show the influence mechanism of hindrance stress on employees’ innovative behavior from the perspective of individual psychology.

In the 1980s, Bandura formally proposed social cognition theory (Bandura, 1985). Its core tenet is that human activities are determined by the interactions of individual behavior, individual cognition, and an individual’s environment. Social cognition theory has been widely used in various fields since it was proposed. According to social cognition theory, the individual characteristics of employees affect their work attitude and behavior. In recent years, with the rise in positive psychology, proactive personality—as an individual positive personality trait—has gradually become a new factor in research (Zhang and Yang, 2017). Research shows that possessing a proactive personality trait can significantly positively predict the innovation ability of employees; that is, individuals with a high degree of proactive personality traits are more innovative and have more new ideas at work than individuals with low degrees of this characteristic (Kim et al., 2009; Zhou et al., 2020).

Person–organization fit theory posits that individual attitudes and behaviors are the result of the interaction between individuals and their environment; organizational climates, as specific organizational situations, obviously play an important role in the connection between organizations and individual innovative behaviors. With the gradual deepening of people’s understanding of innovation, researchers have begun to focus on the impact of soft organizational environments on innovation behavior (Lian et al., 2013). As a feature of organizational climates, the employment relationship atmosphere reflects the overall quality of the rapport between employees and managers. A harmonious employment relationship atmosphere can—to a certain extent—increase employees’ sense of psychological security and job stability. In addition, providing employees with supportive resources may alleviate the negative effect of work stress on employees’ physical and psychological well-being, thereby promoting employees’ innovative behavior (Cao et al., 2020).

In real-life working environments, the innovative behavior of employees is a result of the joint action of various factors. Therefore, this study selected hindrance stress, proactive personality, and the employment relationship atmosphere as representative variables of work characteristics, individual factors, and social situation characteristics, respectively. A multiple linear model was used to empirically analyze the interaction mechanism among hindrance stress, proactive personality, employment relationship atmosphere, and employee innovative behavior. The aim of this research was to enrich the theoretical basis of human resource management and provide guidance for business managers on approaches to effectively stimulating employees’ innovative behavior.

## Theoretical considerations and hypotheses development

### Individual level

Conservation of resources theory suggests that individuals always strive to obtain and maintain resources they consider valuable (Hobfoll, 1989). When employees regard work stress as hindrance, it is difficult for them to see beyond the problem, and they will regard the scenario as a threat to their personal growth and job performance (Lepine et al., 2005). Such stress often consumes significant time and energy, leading individuals to avoid the problem or give up altogether (Pearsall et al., 2009)—neither of which is conducive to fostering innovative behavior. Similarly, the study by Rodell and Judge (2009) showed that blocking stressors are negatively correlated with employees’ organizational citizenship behavior. Moreover, another study demonstrated that employees’ innovative behavior is a component of out-of-role behavior and organizational citizenship behavior (Liu and Shi, 2009). In addition, the difficulties caused by hindrance stress are challenging for employees to overcome through their own efforts and prevent employees from acquiring task-related knowledge and skills in a timely manner. As a result, employees’ enthusiasm decreases in the face of such stress, and they may even assume a passive attitude in dealing with workplace problems. With this in mind, we propose the following hypotheses for investigation in this study:

*H1*: Hindrance stress negatively affects employees’ innovative behavior.

The concept of the proactive personality trait was proposed by Bateman and Crant, who believed that employees with proactive personalities would not be constrained by their situation and would actively create opportunities to change or influence external environmental factors in order to achieve their goals (Bateman and Crant, 1993). Enhancing job control and autonomy through positive change (Li et al., 2014) is an important predictor of innovative behavior and creativity (Liang et al., 2019). Individuals who exhibit a high degree of proactive personality traits tend to show strong autonomy and initiative (Zhang et al., 2017). They actively seek opportunities and take action, and once they take action, they persevere until significant changes are achieved (Crant, 2000). Individual initiative and innovative behavior usually manifest in the identification of problems and opportunities and in the generation of novel ideas and innovative working methods (Shalley et al., 2000). Furthermore, proactive behavior is among the nine core work role behaviors that Griffin et al. identified (Griffin et al., 2007). Individuals with a high degree of proactive personality traits are more willing to take actions to develop new working methods to control and improve their working environment, allowing them to acquire new knowledge, develop new capabilities, undertake constructive changes, correct existing problems, and achieve individual and organizational goals through self-conscious and spontaneous innovation. Therefore, individuals with a high degree of proactive personality traits usually play the role of “pathfinder,” actively affecting the world around them. These findings inform the following two hypotheses proposed in this study.

*H2*: Proactive personality positively affects employees’ innovative behavior.

Work stress consumes a considerable amount of employees’ resources. According to resource-conservation theory, if employees are unable to protect their resources or obtain new ones, they are likely to suffer from excessive depletion of emotional reserves, which will negatively affect their job performance. A proactive personality is a kind of personal resource that allows individuals to create opportunities to acquire other valuable resources. Studies have shown that employees with proactive personalities are good at improving their working environment by seeking information and identifying opportunities (Crant, 2000), thereby bolstering their ability to solve problems at work. In addition, employees with proactive personalities manage their relationships with their superiors well, and they receive more support from leaders (Li et al., 2010), thereby acquiring the resources they need to achieve work goals. Negative and less proactive employees are more likely to passively adapt to their environment rather than actively create opportunities to change it. Therefore, when confronted with hindrance stress, individuals with strong proactive personalities will actively create opportunities to change or influence external environmental factors to achieve their goals (Bateman and Crant, 1993) and strengthen their own control over their work (Fuller and Marler, 2009). By contrast, employees with weak proactive personality traits do not actively create or conserve resources; rather, they consume additional emotional resources to maintain equilibrium, but their efforts further deplete their existing resources, thus inhibiting their innovative behavior. Based on this, the following hypothesis was proposed:

*H3*: A proactive personality plays a moderating role in the relationship between hindrance stress and employees’ innovation behavior.

### Organizational level

The employment relationship atmosphere refers to employees’ perception of the overall relationship between managers and employees, which reflects the level of exchange between the two parties and, to a certain extent, the degree to which employee’s feel valued (Ma et al., 2020). In a positive workplace environment, employees feel heard and respected and are willing to freely express their ideas, which enhances their enthusiasm for innovation. Furthermore, in a positive workplace environment, individuals internalize external rules to a high degree and pay attention to the best interests of the organization rather than their own (Reagans and Mcevily, 2003). In other words, a good employment relationship atmosphere will produce motivated, loyal, and high-performing employees, who will try their best to complete their work responsibilities for the company (Daniel, 2003), thus enhancing employee initiative. Finally, an organizational atmosphere of trust, openness, and support can effectively promote employee learning and motivate self-improvement while fostering ongoing learning within the organization (Pedler et al., 1993) and promote the exchange and integration of knowledge (Collins and Smith, 2006). Thus, organizations can effectively encourage the innovation ability of employees.

*H4*: The employment relationship atmosphere has a positive impact on employees’ innovative behavior.

According to the theory of resource conservation, individuals have a tendency to conserve, protect, and acquire resources, with the actual or potential loss of resources causing tension for individuals. Because of its uncontrollability and ambiguity, hindrance stress is considered detrimental to employee innovation. However, when an organization promotes a harmonious employment relationship atmosphere, employees are better able to cognitively evaluate stressors, and this helps employees overcome the negative emotions brought on by hindrance stress (Li and Peng, 2014), which in turn fosters their innovative behavior (Li and Peng, 2014; Loi et al., 2014). In addition, when the employment relationship atmosphere is positive, there is mutual trust and respect between employees and their supervisors. Good stewardship of the employer–employee relationship will strengthen the interaction between the two sides. Encouraging supervisors to recognize employees’ work and help employees obtain information and emotional support not only bolsters employees’ commitment and increases their job satisfaction, it also improves employees’ optimism and thus their ability to cope with work pressure, which in turn stimulates their proactive and innovative behaviors (Loi et al., 2014; Breevaart et al., 2015).

*H5*: The employment relationship atmosphere plays a moderating role in the relationship between hindrance stress and employees’ innovative behavior.

**Figure 1:**
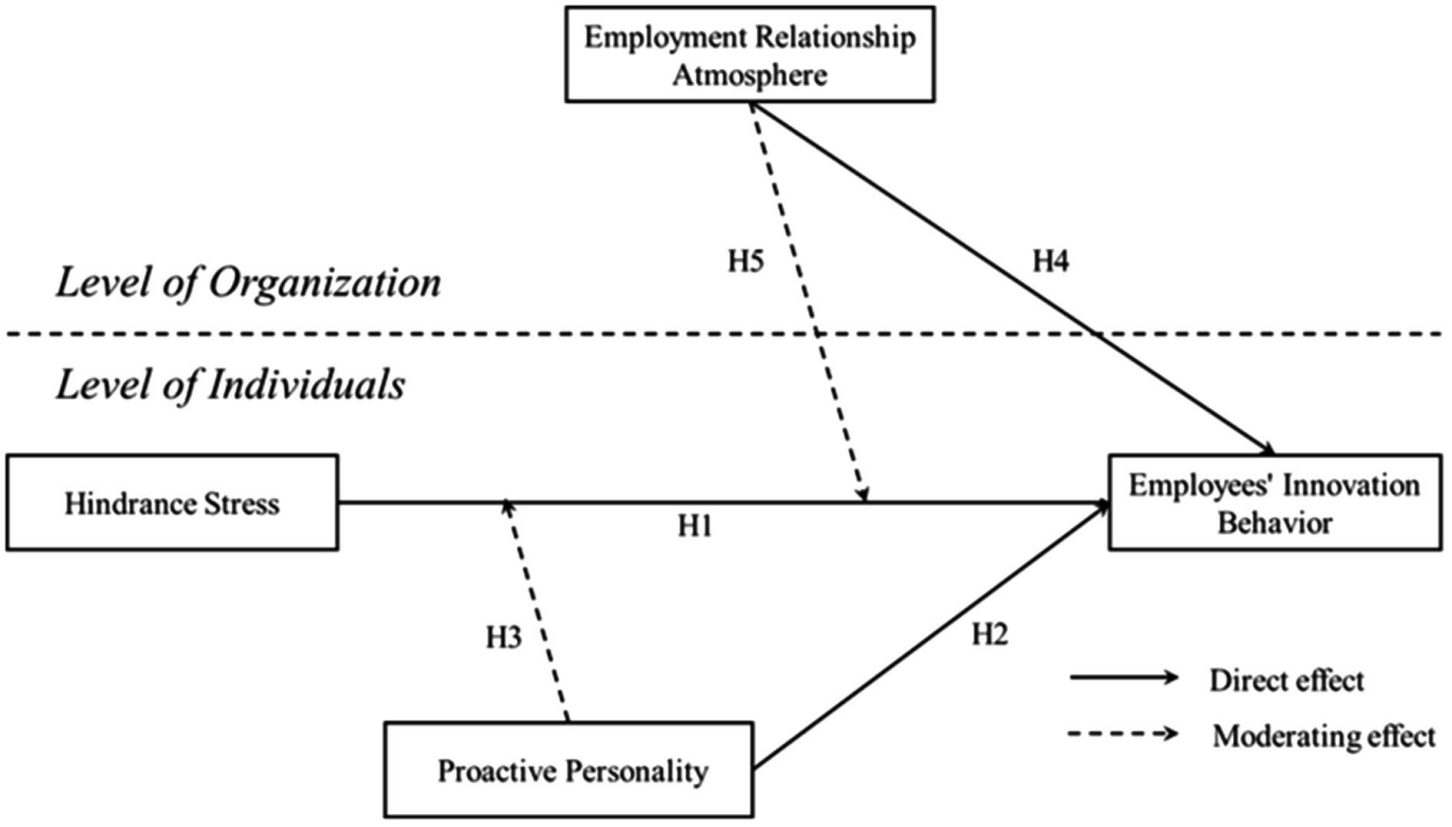
Research model.

Based on the above analysis, this study proposes a theoretical research model as shown in Figure 1.

## Materials and methods

### Pretest

A pretest plays a critical role in identifying problems with question. Pretests typically involve a few experienced interviewers conducting 25–75 interviews (Oksenberg et al., 1991). In this study, a total of 50 questionnaires were sent out, and 42 returns were valid. For the measurement of variables, two indicators might be fine, three indicators are better, four indicators are best, and anything more gravy (Kenny, 1979). Based on this and the research results of relevant scholars, combined with the actual work of employees, in this study, we modified the measurement indicators of relevant variables to make them more concise and in line with the research scenario. For example, for the item of hindrance stress, the factor load of the item “political factors, not performance, are the key factors affecting organizational decisions” was lower than 0.6, so it was deleted; among the items of proactive personality, the items “If I see something I do not like, I fix it” and “I excel at identifying opportunities” were close to the other items, and the factor loading was lower than 0.6, so they were deleted;among the items about employees’ innovation behavior, the item “I am a person with innovative spirit” was deleted because it could—to a certain extent—induce participants to give answers in line with social expectations.

In the analysis of the pretest questionnaire, Cronbach’s α for each subscale was between 0.774 and 0.861, and the explanatory ability of each variable for the topic also met the requirements, indicating that the questionnaire had good reliability and validity, and could be used for formal investigation and research.

### Data collection

For sample size requirements, Cora and Hox suggested that each group should have 5 samples, and with 50 groups, the non-coverage drops to about 7.3%. This is clearly different from the nominal 5%, but in practice, this is acceptable (Cora and Hox, 2005). We selected 60 companies in China, Philippine, Kazakhstan, New Zealand, and the United Kingdom to recruit participants for the study, and each company invited 5–8 people to fill in the questionnaire. Such a sample size meets the above requirements.

We collected data using online questionnaires. The first section of the questionnaire comprised scaled instruments of the selected variables. A letter was attached to the questionnaire to describe the study’s objectives and to assure participants of the confidentiality of the responses. We then sent the questionnaire links to the participants and asked them to complete the questionnaire. Before completing the questionnaire, the participants fully understood the meaning of its content and confirmed their understanding of both the significance of the questionnaire and the use of the data. Participants were compensated with USD 2 for finishing each survey.

Of 400 questionnaires, 349 were returned. After removing invalid questionnaires, 331 questionnaires from 57 companies were retained. Among the 57 companies, there were 18 supermarkets, 16 restaurants, 10 hotels, 8 hospitals, and 5 factories. Of the 331 respondents, 189 were male and 142 were female. The average age was 37.6 years. A total of 59 participants held graduate degrees or above, 151 held university degrees, and 121 held high school degrees or below.

### Common method bias test

In this study, procedural control was adopted to reduce the common method bias by avoiding respondents’ guesses about the subjects being measured, balancing the sequence effect of items, and protecting respondents’ anonymity. In addition, the Harman single-factor test was used to detect the degree of common method variation of the data. According to the results of SPSS 24.0, the variance explanation rate of the first factor was 32.48%. Since it did not account for half of the total variation, this meant that the common method deviation of the data in this study was statistically well controlled.

### Measurement of variables

Our assessment of hindrance stress, as adapted from Cavanaugh et al. (2000) and Ren and Zhang (2015), and Zhang et al. (2018), included 4 items, for example: “My work tasks and goals are not clear” and “I often get different and conflicting job requests.” Our assessment of proactive personality traits, as adapted from Seibert et al. (1999), included 4 items, for example: “Wherever I have been, I have been a powerful force for constructive change” and “I am always looking for better ways to do things.” Our assessment of employment relationship atmosphere, as adapted from Ngo et al. (2008) and Ma et al. (2020), included 5 items, for example: “I can give full play to my knowledge and skills in the company” and “When encountering problems, I can communicate and discuss with leaders and colleagues honestly.” Our assessment of employees’ innovation behavior, as adapted from Bani et al. (2018) and Wang et al. (2019), included 4 items, for example: “I often generate new ideas and creative ideas” and “I often find new ways to solve problems in my work.”

All items were measured on a 7-point Likert scale, anchored at 1—strongly disagree; 7—strongly agree. All items were translated into different languages by professionals so that participants could fill it out easily.

### Analysis tools

In this study, SPSS 24.0, Mplus 8.0, and HLM 6.08 were used for data analysis and processing.

## Results

### Reliability and validity

The results of the reliability and validity analyses of variables are shown in Table 1. The factor load of each measurement item was above 0.669, and there was no negative error variation, which met the inspection standard. The C.R. value of each variable was between 0.829 and 0.874, and the AVE was above 0.550, indicating good reliability and convergent validity. The correlation coefficients between variables are shown in Table 2. The square root value of AVE was greater than the correlation coefficient between variables, indicating that the model has good differential validity.

**Table 1:** Results of confirmatory factor analysis and reliability and validity tests.

Dim	Item	Parameters of significant test				Item reliability	Composite reliability	Convergence validity
		Estimate	S.E.	Est./S.E	*p*-value	SMC	C.R.	AVE
HS	HS1	0.717	0.021	34.143	***	0.514	0.829	0.550
	HS2	0.825	0.013	63.462	***	0.681		
	HS3	0.735	0.018	40.833	***	0.540		
	HS4	0.682	0.013	52.462	***	0.465		
PP	PP1	0.751	0.015	50.067	***	0.564	0.834	0.558
	PP2	0.669	0.013	51.462	***	0.448		
	PP3	0.747	0.017	43.941	***	0.558		
	PP4	0.813	0.014	58.071	***	0.661		
ERA	ERA1	0.757	0.021	36.048	***	0.573	0.874	0.583
	ERA2	0.728	0.023	31.652	***	0.530		
	ERA3	0.679	0.013	52.231	***	0.461		
	ERA4	0.811	0.016	50.688	***	0.658		
	ERA5	0.832	0.019	43.789	***	0.692		
EIB	EIB1	0.835	0.013	64.231	***	0.697	0.858	0.604
	EIB2	0.821	0.016	51.313	***	0.674		
	EIB3	0.735	0.013	56.538	***	0.540		
	EIB4	0.709	0.025	28.057	***	0.503		

HS, Hindrance Stress; PP, Proactive Personality; ERA, Employment Relationship Atmosphere; EIB, Employees’ Innovation Behavior. ***p < 0.001; **p < 0.01; *p < 0.05.

**Table 2:** Variables’ correlation coefficient and the square root of AVE.

Dim	Convergence validity	Discriminate validity			
	AVE	HS	PP	ERA	EIB
HS	0.550	**0.742**			
PP	0.558	0.167	**0.747**		
ERA	0.583	−0.218	0.274	**0.764**	
EIB	0.604	−0.538	0.347	0.501	**0.777**

The bold diagonal font is the square root value of AVE, and the lower triangle is Pearson correlation coefficient.

### Basic characteristic test

The employment relationship atmosphere in this study belongs to the shared construct, data were collected from individuals within different companies, so $r_{wg}$ was used as an indicator to test the appropriateness of aggregating variables to the organizational level. In practice, $r_{wg}$ greater than 0.70 is generally considered acceptable (Zohar, 2000). In this study, the average $r_{wg}$ of employment relationship atmosphere was 0.813, which met the relevant requirements.

### Hypothesis testing

#### Null model

The null model, also known as the empty model, is the most basic framework and the starting point of hierarchical linear model analyses. The relevant models are described below. The null model was used for variance components analysis. The results show that the within-group component (*σ*^2^) was 0.235, the between-group component (*τ*_00_) was 0.171, and *ICC*1 was 0.421; according to Cohen (1988), these values showed high correlation, indicating that the differences between groups could not be ignored, and was is not suitable to use a general regression model for analysis.

Level 1: $EIB_{ij}=\beta_{0j}+r_{ij.}$

Level 2: $\beta_{0j}=\gamma_{00}+u_{0j}$

Mixed Model: $EIB_{ij}=\gamma_{oo}+u_{oj}+r_{ij}$

#### Random coefficients regression model

Only the first level of the random coefficients regression model had independent variables, and the second level was a null model. The regression models of the first level—including the intercept terms and the slope terms—were set as random effects in the second level. This analysis model was designed to test whether the intercept and slope of the first level regression model existed. The correlation analysis model is shown below. In this model, both the hindrance stress and the proactive personality factors were group-centered. The results are shown in Table 3: H1, H2, and H3 were verified. Meanwhile, *σ*^2^ was 0.157, with a decrease of 0.078; this indicates that, when the hindrance stress, proactive personality, and their interaction are added to the first level, the proportion of variance within the group decreased by 33.2%, and other factors still existed in the first level.

**Table 3:** Results of random coefficients regression model.

	Effect	Standard coefficient error	Approx. T-ratio	*p*-value	Hypothesis (Y/N)
γ_00_	3.837	0.051	75.504	***	
γ_10_	0.140	0.045	3.093	**	H2(Y)
γ_20_	−0.430	0.047	−9.138	***	H1(Y)
γ_30_	0.063	0.019	3.178	**	H3(Y)

***p < 0.001; **p < 0.01; *p < 0.05.

**Figure 2:**
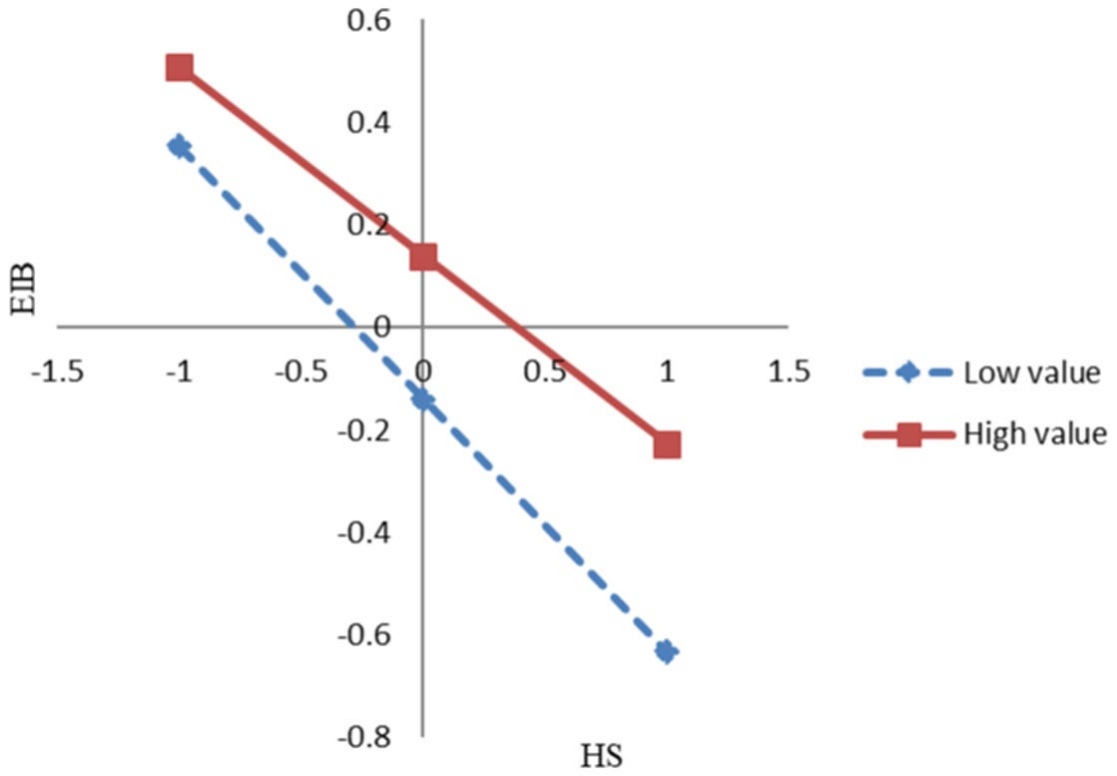
The moderating effect of proactive personality (PP).

A simple slope test shows that the gradient of the slope for low proactive personality was −0.493 (*t* = 3.532, *p* < 0.001), indicating that the gradient of the slope for low proactive personality was significant. In the same way, the gradient of the slope for high proactive personality was −0.367 (*t* = 2.854, *p* < 0.01), indicating that the gradient of the slope for high proactive personality was also significant. Figure 2 shows the moderating effect of proactive personality. By comparison, the gradient of the slope for the high proactive personality was larger than that of the low proactive personality. This means that the higher proactive personality the employee is, the negative impact of hindrance stress on employee innovation behavior is weaker.

Level 1: $\begin{array}{l} EIB_{ij}=\beta_{0j}+\beta_{1j}\left(PP_{ij}-{\bar{PP}}_{.j}\right)+\\ \beta_{2j}\left(HS_{ij}-{\bar{HS}}_{.j}\right)+\\ \beta_{3j}\left(PP_{ij}-{\bar{PP}}_{.j}\right)\left(HS_{ij}-{\bar{HS}}_{.j}\right)+r_{ij} \end{array}$

Level 2: $\beta_{0j}=\gamma_{00}+u_{0j}$ ; $\beta_{1j}=\gamma_{10}+u_{1j}$ ; $\beta_{2j}=\gamma_{20}+u_{2j}$ ; $\beta_{3j}=\gamma_{30}+u_{3j}$

Mixed Model: $\begin{array}{l} EIB_{ij}=\gamma_{00}+\gamma_{10}\left(PP_{ij}-{\bar{PP}}_{.j}\right)+\\ \gamma_{20}\left(HS_{ij}-{\bar{HS}}_{.j}\right)+\\ \gamma_{30}\left(PP_{ij}-{\bar{PP}}_{.j}\right)\left(HS_{ij}-{\bar{HS}}_{.j}\right)+\\ u_{0j}+u_{1j}\left(PP_{ij}-{\bar{PP}}_{.j}\right)+\\ u_{2j}\left(HS_{ij}-{\bar{HS}}_{.j}\right)+\\ u_{3j}\left(PP_{ij}-{\bar{PP}}_{.j}\right)\left(HS_{ij}-{\bar{HS}}_{.j}\right)+r_{ij} \end{array}$

#### Intercepts as outcomes model

In order to further understand the main effects of the second-level employment relationship atmosphere, the intercepts were analyzed as an outcomes model, and the relevant analysis model is shown here. In this model, the atmosphere of employment relationship is dealt with in a grand centered way. The results are shown in Table 4—H4 was verified. At the same time, *τ_00_* was 0.129, reduced by 0.042. This indicated that, when the employment relationship atmosphere was added to the second level, the proportion of variance reduction between groups was 24.6%, and other influencing factors remained at the second level.

**Table 4:** Results of intercepts as outcomes model.

	Effect	Standard coefficient error	Approx. T-ratio	*p*-value	Hypothesis (Y/N)
γ_00_	3.819	0.048	80.185	***	
γ_01_	0.379	0.073	5.168	***	H4(Y)

***p < 0.001; **p < 0.01; *p < 0.05.

Level 1: $EIB_{ij}=\beta_{0j}+r_{ij}$

Level 2: $\beta_{0j}=\gamma_{00}+\gamma_{01}\left(ERA_{j}-{\bar{ERA}}_{.}\right)+u_{0j}$

Mixed Model: $EIB_{ij}=\gamma_{oo}+\gamma_{01}\left(ERA_{j}-{\bar{ERA}}_{.}\right)+u_{oj}+r_{ij}$

#### Slope as outcomes model

As mentioned above, since the slope terms of the random coefficients regression model were significantly different, in order to further understand whether the slope variance component could be explained by the second-level employment relationship atmosphere, it was necessary to continue the analysis of the slope as an outcomes model. The correlation analysis model is shown below. The results are shown in Table 5—H5 was verified.

**Table 5:** Results of slope as outcomes model.

	Effect	Standard coefficient error	Approx. T-ratio	*p*-value	Hypothesis (Y/N
γ_00_	3.596	0.163	22.062	***	
γ_01_	0.363	0.075	4.815	***	
γ_10_	0.137	0.044	3.093	**	
γ_20_	−0.429	0.041	−10.458	***	
γ_21_	0.132	0.062	2.161	*	H5(Y)
γ_30_	0.061	0.019	3.119	**	

***p < 0.001; **p < 0.01; *p < 0.05.

**Figure 3:**
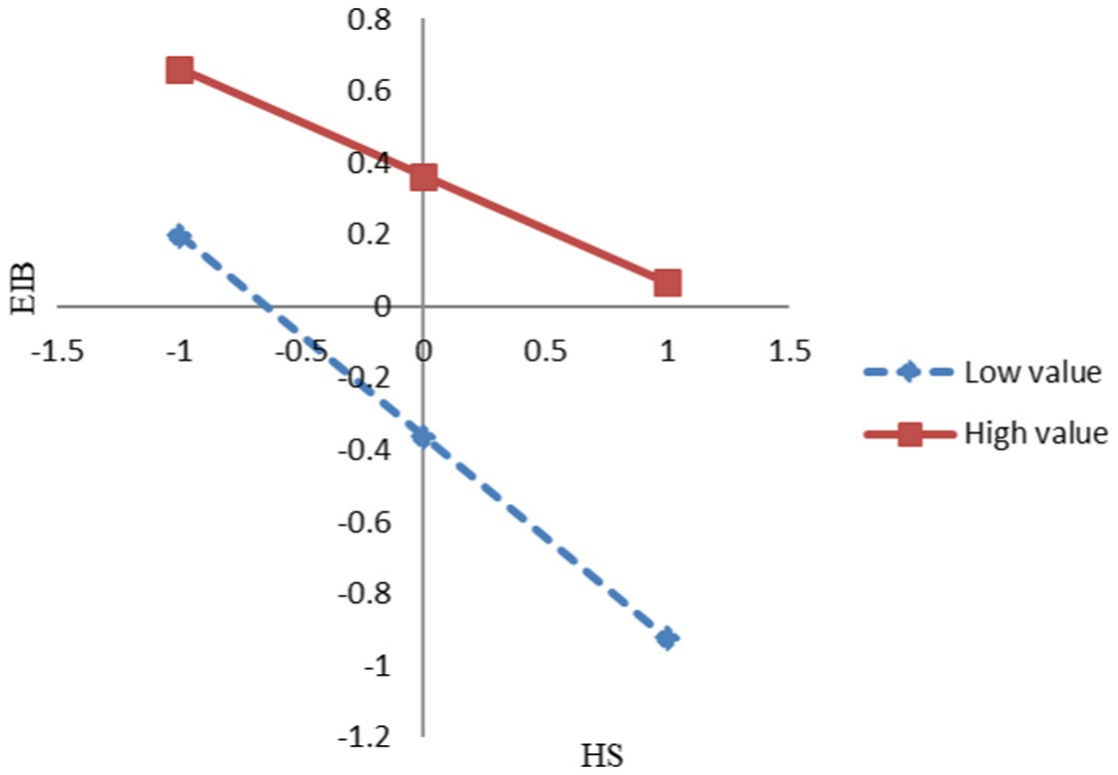
The moderating effect of employment relationship atmosphere (ERA).

A simple slope test shows that the gradient of the slope for low employment relationship atmosphere was −0.561 (*t* = 2.362, *p* < 0.05), indicating that the gradient of slope for low employment relationship atmosphere was significant. Moreover, the gradient of the slope for high employment relationship atmosphere was −0.397 (*t* = 2.017, *p* < 0.05), indicating that the gradient of the slope for high employment relationship atmosphere was also significant. Figure 3 shows the moderating effect of the employment relationship atmosphere. By comparison, the gradient of the slope for high employment relationship atmosphere was larger than that of low employment relationship atmosphere. This means that, the better an employee’s perception of the employment relationship atmosphere, the weaker the negative impact of hindrance stress on that employee’s innovation behavior.

Level 1: $\begin{array}{l} EIB_{ij}=\beta_{0j}+\beta_{1j}\left(PP_{ij}-{\bar{PP}}_{.j}\right)+\\ \beta_{2j}\left(HS_{ij}-{\bar{HS}}_{.j}\right)+\\ \beta_{3j}\left(PP_{ij}-{\bar{PP}}_{.j}\right)\left(HS_{ij}-{\bar{HS}}_{.j}\right)+r_{ij} \end{array}$

Level 2: $\beta_{0j}=\gamma_{00}+\gamma_{01}\left(ERA_{j}-{\bar{ERA}}_{.}\right)+u_{0j};$ $\beta_{1j}=\gamma_{10}+u_{1j};$

$\beta_{2j}=\gamma_{20}+\gamma_{21}\left(ERA_{j}-{\bar{ERA}}_{.}\right)+u_{2j}$ ;

$\beta_{3j}=\gamma_{30}+u_{3j}$

Mixed Model: $\begin{array}{l} EIB_{ij}=\gamma_{00}+\gamma_{01}\left(ERA_{j}-{\bar{ERA}}_{.}\right)+\\ \gamma_{10}\left(PP_{ij}-{\bar{PP}}_{.j}\right)+\\ \gamma_{20}\left(HS_{ij}-{\bar{HS}}_{.j}\right)+\\ \gamma_{21}\left(ERA_{j}-{\bar{ERA}}_{.}\right)\left(HS_{ij}-{\bar{HS}}_{.j}\right)+\\ \gamma_{30}\left(PP_{ij}-{\bar{PP}}_{.j}\right)\left(HS_{ij}-{\bar{HS}}_{.j}\right)+\\ u_{0j}+u_{1j}\left(PP_{ij}-{\bar{PP}}_{.j}\right)+\\ u_{2j}\left(HS_{ij}-{\bar{HS}}_{.j}\right)+\\ u_{3j}\left(PP_{ij}-{\bar{PP}}_{.j}\right)\left(HS_{ij}-{\bar{HS}}_{.j}\right)+r_{ij} \end{array}$

## Discussion

### Theoretical contribution

This study analyzed the mechanisms between hindrance stress and employee innovation behavior.

In the workplace, work pressure is one of the most common organizational environmental stimuli, affecting employees’ cognition, stimulating corresponding motivation, and leading to corresponding behaviors (Zhao and Yang, 2020). If employees deem stressors to be a hindrance, this has a negative impact on their behavior (Cavanaugh et al., 2000). This study firstly verified the negative impact of hindrance stress on employees’ innovative behavior. Second, in real life, the innovation performance of different employees in the same organization will not be the same, which indicates that personality characteristics have a certain impact on the innovation behavior of employees. This study found that proactive personality, as an important personality trait, was positively correlated with employees’ innovative behavior, and played a moderating role in the relationship between hindrance stress and employees’ innovative behavior. Third, Individuals in a specific environment have different perceptions of the environment, producing different behaviors (Lepine et al., 2005). This study reveals that the employment relationship atmosphere positively correlated with employees’ innovative behavior, playing a moderating role in the relationship between hindrance stress and employees’ innovative behavior.

Previous studies mainly focus on the individual-level factors in this discourse, ignoring the cross-level influence of multi-level factors on employee innovation behavior (Yan et al., 2019). In such studies, when processing the relevant data, variables at the organizational level were transformed into characteristics at the individual level, without using multi-level analysis technology. This study comprehensively considered variables at the individual level and organizational level and used a hierarchical linear model method to analyze it. This may help scholars of innovation behavior to further understand the influence of variables at different levels on employees’ innovative behavior.

### Practical implications

1. Organizations should pay attention to the negative impact of hindrance stress on employees’ innovative behavior. The first way to achieve this is through individual interviews and other methods, where the factors that lead to hindrance stress can be regularly assessed, targeting the root cause of the problems. The second way to achieve this is to establish an open communication platform for employees within the organization to create effective lines of communication, thereby hindering the negative impact of stress on employees. Thirdly, performance assessment indicators should be formulated at different levels according to position and rank to optimize employees’ innovative behaviors.
2. Organizations should give full attention to the positive impact of proactive personality traits on employees’ innovative behavior. Beginning with the recruitment stage, organizations can select employees with proactive personality traits according to their specific situation of innovation practice. In addition, according to the individual characteristics of employees, organizations should clarify employees’ work roles with targeted work objectives and work content, and a reasonable arrangement of work responsibilities and requirements. Moreover, providing ongoing work-related training to existing employees will help them cultivate self-confidence and positivity, enhance resilience in the face of hindrance stress, and thus improve their enthusiasm and initiative in innovative activities.
3. The nature of the employment relationship atmosphere is an important measure of the quality of the relationship between employees and employers and the sustainable development of the organization. A harmonious employment relationship atmosphere can enhance the psychological security and job commitment of employees and stimulate their innovative behavior. Managers at all levels should pay attention to creating a positive atmosphere that fosters sustainable employment relations. This can be achieved by maintaining an open dialogue with employees at different levels so that employees receive equal access to information and resource support, so as to promote the continuous development of employees’ innovative activities.

### Limitations and future directions

The data used in this paper are cross-sectional, which may negatively affect judgments of causality. Future studies should collect data at different time points through longitudinal tracking to improve the credibility. Although the measurement of variables in the study has high reliability and validity, different cultural backgrounds may have a certain impact on the research results. The issue of cultural differences needs to be considered in future research.

## Data availability statement

The original contributions presented in the study are included in the article/supplementary material, further inquiries can be directed to the corresponding author.

## Author contributions

JF is responsible for research design, theoretical review, and data processing. YF and LY were responsible for questionnaire design and data collection. SM was responsible for writing the discussion section. All authors contributed to the article and approved the submitted version.

## Funding

This research was supported by the Doctoral Research Foundation of Pingdingshan University (PXY-BSQD-2022041).

## Conflict of interest

The authors declare that the research was conducted in the absence of any commercial or financial relationships that could be construed as a potential conflict of interest.

## Publisher’s note

All claims expressed in this article are solely those of the authors and do not necessarily represent those of their affiliated organizations, or those of the publisher, the editors and the reviewers. Any product that may be evaluated in this article, or claim that may be made by its manufacturer, is not guaranteed or endorsed by the publisher.
